# Oocyte Age‐Dependent DNA Damage Can Be Reverted by the DNA Repair Competent Karyoplasm of Young Oocytes

**DOI:** 10.1111/acel.70300

**Published:** 2025-11-15

**Authors:** Nataliia Dudko, Tereza Ilcikova, Natalie Novotna, Marta Czernik, Pasqualino Loi, Josef Fulka, Pritha Bhattacharjee, Raffaella Santoro, Helena Fulka

**Affiliations:** ^1^ Institute of Experimental Medicine of the Czech Academy of Sciences Prague Czech Republic; ^2^ University of Teramo Teramo Italy; ^3^ Institute of Animal Science Prague Czech Republic; ^4^ University of Zurich Zurich Switzerland

**Keywords:** ageing, chromosomal aberrations, DNA damage, oocyte

## Abstract

Mammalian fully grown oocytes are believed to exhibit a weakened DNA damage response, leading to the accumulation of substantial levels of DNA damage and increased frequency of aneuploidies in an age‐dependent manner. These hallmarks of reproductive ageing are generally presumed to be irreversible by rendering the oocyte chromosome complement incompatible with development. To test whether this is indeed true, we performed a series of germinal vesicle (GV) transfers between oocytes from females of late breeding/post‐breeding age and oocytes from young animals. Our results show that age‐associated DNA damage can be effectively suppressed: introducing the GVs of advanced‐maternal‐age (AMA) oocytes into DNA repair‐competent cytoplasts generated by selective enucleation (SE) of young oocytes effectively suppresses the signs of age‐dependent DNA damage. This is accompanied by a partial recovery of the chromatin dynamics and, surprisingly, a higher fidelity of chromosome segregation. By dissecting the GV fractions, we show that the ability to sense and repair DNA is linked to the free, non‐chromatin‐bound nuclear factors but not the oocyte nucleolus. Finally, we show that the overall improved state of the reconstructed oocytes is accompanied by enhanced full‐term development. Therefore, contrary to popular belief, our results show that the age‐associated decline in oocyte quality can be effectively mitigated, opening new possibilities for cell‐based oocyte therapy.

## Graphical abstract

As females age, oocyte chromosomal aberrations and DNA damage increase in frequency; this condition is believed to be irreversible. By exposing the aged oocyte nuclei to nuclear factors from young oocytes, the negative effects can be effectively suppressed, restoring their developmental competence.

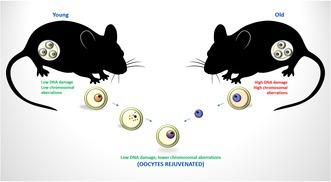

As females age, their reproductive ability declines. This phenomenon has been documented in various mammalian species, ranging from rodents to humans (Holinka and Finch 1981; Short et al. 1993), and is manifested in a reduced oocyte quality, higher frequency of aneuploidies, and miscarriages. These translate to age‐associated infertility. The decline in oocyte quality represents a prominent factor: Using oocytes from healthy young donors effectively mitigates the maternal age effect of recipients, leading to successful pregnancies and deliveries (Paulson et al. 1997; Check et al. 2011; Clain et al. 2022).

One of the factors affecting oocyte quality might be high levels of DNA damage (Sharma et al. 2024; Sun et al. 2024), which were implicated as a direct cause of aneuploidies (Mayer et al. 2016; Sun et al. 2024). Although it has been proposed that oocytes exhibit a weakened DNA damage response (Sun et al. 2024), it is still not entirely clear why and to what extent DNA damage accumulates in advanced maternal age (AMA) oocytes. In yeast, nucleoli as well as cohesin have been shown to play a direct role in DNA repair by organising the spontaneous DNA lesions (Dion et al. 2013). Along those lines, the irreversible REC8 cohesin depletion, which is typical for AMA oocyte chromatin (Chiang et al. 2010), was recently reported as one of the main drivers of DNA damage accumulation (Sharma et al. 2024). Changes to nucleoli and ribosome biogenesis in growing oocytes with female ageing have also been described (Duncan et al. 2017). However, whether chromatin state is decisive for the overall developmental competence of oocytes remains unknown.

## 1 DNA Damage Increases in Oocytes With Female Age

To understand the extent of age‐dependent DNA damage, we analysed oocyte chromosomes of mice of two different age categories: The control group consisted of young adult animals 8–12 weeks of age (young, Y), whereas the experimental group, late breeding and/or post‐breeding age, was in the range of 44–53 weeks (advanced maternal age, AMA).

**FIGURE 1:**
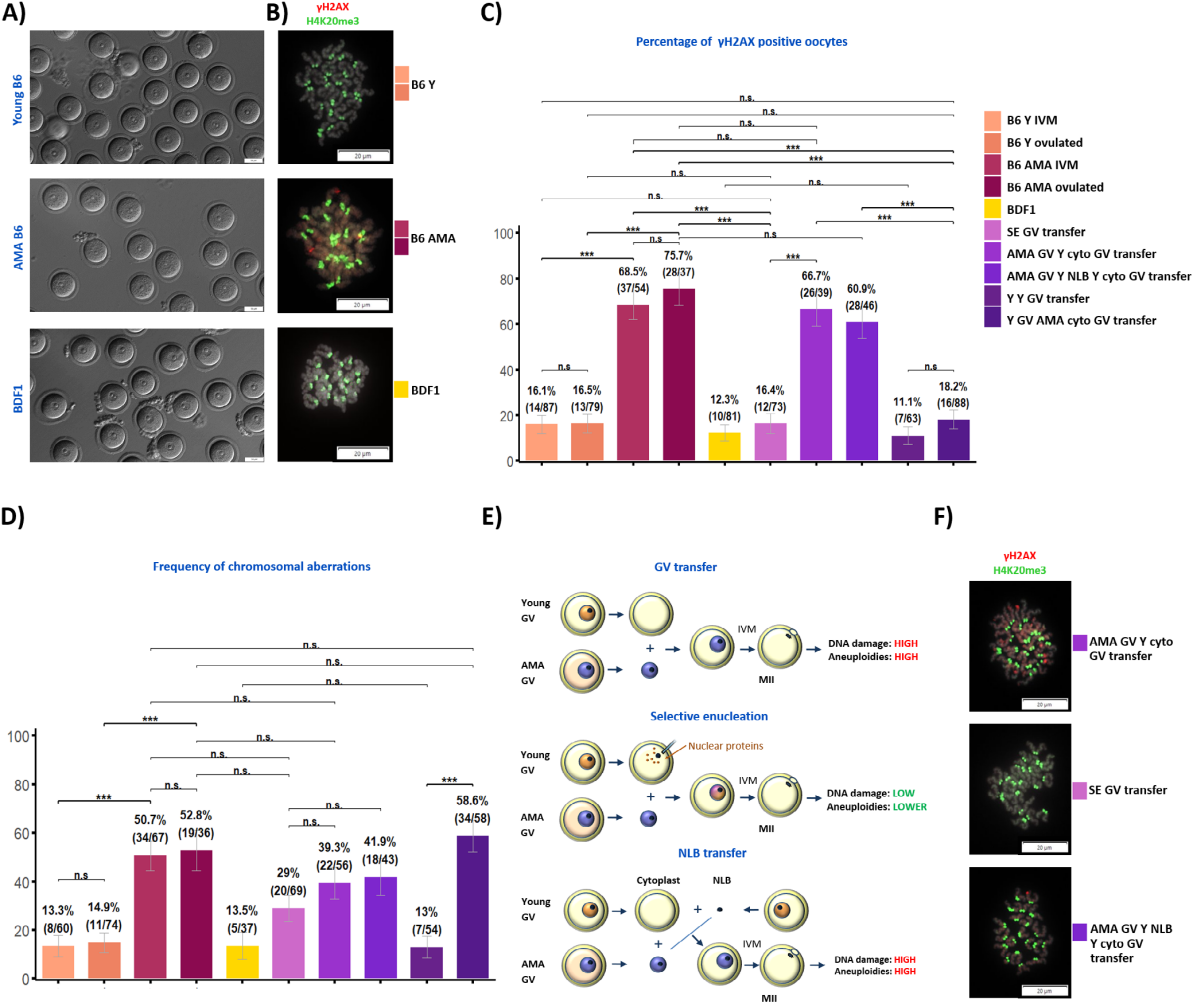
The appearance of oocytes, the frequency of DNA damage, chromosomal aberrations and the experimental scheme of GV transfer experiments. (A) The morphology of isolated germinal vesicle (GV) oocytes. Except for the lower number, the appearance of oocytes retrieved from AMA C57Bl/6J (B6) females is indistinguishable from oocytes recovered from young (Y) animals and young BDF1 oocytes (scale bar: 50 μm). (B) In contrast to metaphase II (MII) oocytes from young animals, AMA oocytes exhibit signs of DNA damage. The chromosomes were labelled for pericentric heterochromatin (histone H4K20me3, green) and γH2A.X (red; DAPI‐grey) (scale bar: 20 μm). (C) The quantification of chromosome spreads exhibiting γH2A.X foci. The data were evaluated by Fisher's exact test (n.s., not significant; ***, statistically significant, see methods for details). The plots shows the aggregated data for the following groups: AMA GV Y cyto GV transfer, oocytes reconstructed by combining AMA GV and young (Y) BDF1 cytoplasm; AMA GV Y NLB Y cyto GV transfer, oocyte reconstructed from AMA GV, young (Y) oocyte nucleolus (nucleolus‐like body, NLB) and young (Y) BDF1 cytoplasm; B6 AMA IVM, B6 advanced maternal age in vitro matured oocytes; B6 AMA ovulated, B6 advanced maternal age in vivo ovulated oocytes; B6 Y IVM, B6 young in vitro maturation; B6 Y ovulated, B6 young in vivo ovulated; BDF1 MII, BDF1 in vivo ovulated oocytes; SE GV transfer, reconstructed oocytes composed of AMA GV and cytoplasts obtained from BDF1 oocytes by selective enucleation (SE); Y GV AMA cyto GV transfer, oocytes reconstructed from young (Y) GVs and advanced maternal age (AMA) cytoplasm; YY GV transfer, control young (Y) oocytes produced by autologous GV transfer. (D) The frequency of chromosomal aberrations. Numerical alterations as well as premature sister separations were considered as aberrations from the expected metaphase II karyotype. (E) The experimental scheme summarising the GV transfer combinations used and representative chromosomal spreads obtained from the reconstructed oocytes. (F) Representative images of chromosomal spreads of the reconstructed oocytes stained for γH2A.X (red) and pericentric heterochromatin (histone H4K20me3, green) (scale bar: 20 μm).

Although we found no clear morphological difference between young and AMA GV‐stage oocytes (Figure 1A), significantly fewer oocytes were retrieved from AMA females (Figure S1). Despite this, the AMA oocytes mature well in vitro (Figure S2).

The oocytes of both categories were labelled for γH2A.X, a marker of double‐strand DNA breaks, as a DNA damage proxy (Figure 1B). Although the chromosomes from young oocytes rarely contained the γH2A.X signal (14/87; 16.1%), nearly 70% of chromosomal spreads from AMA oocytes showed prominent γH2A.X foci (37/54; 68.5%) (Figure 1C). The number of foci per chromosomal spread was, on average, higher in the AMA group (3.9 vs. 2.9 foci/chromosome spread). As expected, the frequency of chromosome number aberrations and prematurely separated sister chromatids was relatively high in in vitro‐matured AMA metaphase II oocytes (34/67; 50.7%). In young oocytes, most chromosomal spreads contained 20 pairs of joined sister chromatids (chromosomal aberrations were detected in 8/60, 13.3%) (Figure 1D).

As these results can be skewed by isolating oocytes that would not normally be ovulated, we analysed in vivo‐ovulated ones (Figure 1C,D). Here, the frequency of chromosome aberrations was also relatively high in the AMA group (19/36; 52.8% vs. young: 11/74; 14.9%) (Figure 1D). In agreement with the results from the in vitro setup, the in vivo ovulated AMA oocytes also frequently exhibited a γH2A.X signal (28/37; 75.7%), with an average of 2.1 foci/chromosome spread. Therefore, DNA damage does not appear to affect maturation rates, and oocytes with damaged DNA are regularly ovulated by AMA females after hormonal stimulation.

Since changes to GV chromatin organisation and compaction with female age were described (Baumann et al. 2023; Manosalva and González 2010), and the nucleolus as the central element of the 3D genome organisation can be important with respect to DNA damage in centromeric regions (Ogushi et al. 2017), we asked whether differences in the nuclear morphology can be detected. However, as with the external morphology, we found no marked difference between the two age groups as judged by the fraction of centromeres associated with nucleoli (nucleolus‐like bodies; NLBs) (Figure S3). In summary, despite a significantly higher frequency of DNA damage, there seems to be no clear link with gross nuclear morphology.

## 2 Young Fully Grown Oocytes Sense DNA Damage and Are Repair‐Competent

To determine whether this state could be reverted, we performed a series of nuclear transfers (Figure 1E and Figure S4). First, we transferred GVs between AMA and young oocytes. Assuming that the accumulation of DNA damage depends on the state of the nucleus, we expected this to have a limited impact. Indeed, the young oocyte cytoplasm did not reduce the overall number of γH2A.X‐positive AMA chromosomal spreads, and 26/39 of the analysed AMA chromosomal spreads contained at least one γH2A.X focus (“AMA GV‐Y cyto transfer”: 26/39; 66.7%) (Figure 1C,F). This confirms that the DNA repair machinery is confined to the oocyte nucleus, and the repair capacity diminishes with maternal age.

Next, we combined selective enucleation (SE) with GV transfer. In this combination, we tested whether the young repair‐competent karyoplasm can normalise the AMA chromatin. During SE, the GV envelope ruptures, releasing nuclear proteins and organelles that are not tightly bound to the DNA into the cytoplasm; the DNA itself and tightly associated proteins are removed (Fulka et al. 2019; Greda et al. 2006). Surprisingly, the SE cytoplasts effectively suppressed the DNA damage signs in the AMA chromosomes. Of the 73 chromosomal spreads, only 12 contained at least one γH2A.X focus (“SE‐GV transfer”: 12/73; 16.4%) (Figure 1C,F). The frequency of chromosomal aberrations also decreased (20/69; 29%) (Figure 1D).

To further dissect which nuclear component is responsible for the effect, we produced oocytes containing AMA GVs, young cytoplasm, and young nucleoli, but not the soluble young karyoplasm (“AMA GV‐Y NLB‐Y cyto”). Here, 28/46 (60.9%) AMA chromosomal spreads showed signs of DNA damage (Figure 1C,F). The chromosomal aberration rate also did not improve markedly (18/43; 41.9%) (Figure 1D). Therefore, the observed positive effect is not linked to oocyte nucleoli, but to the soluble nuclear fraction, which can potentially alter the AMA chromatin.

**FIGURE 2:**
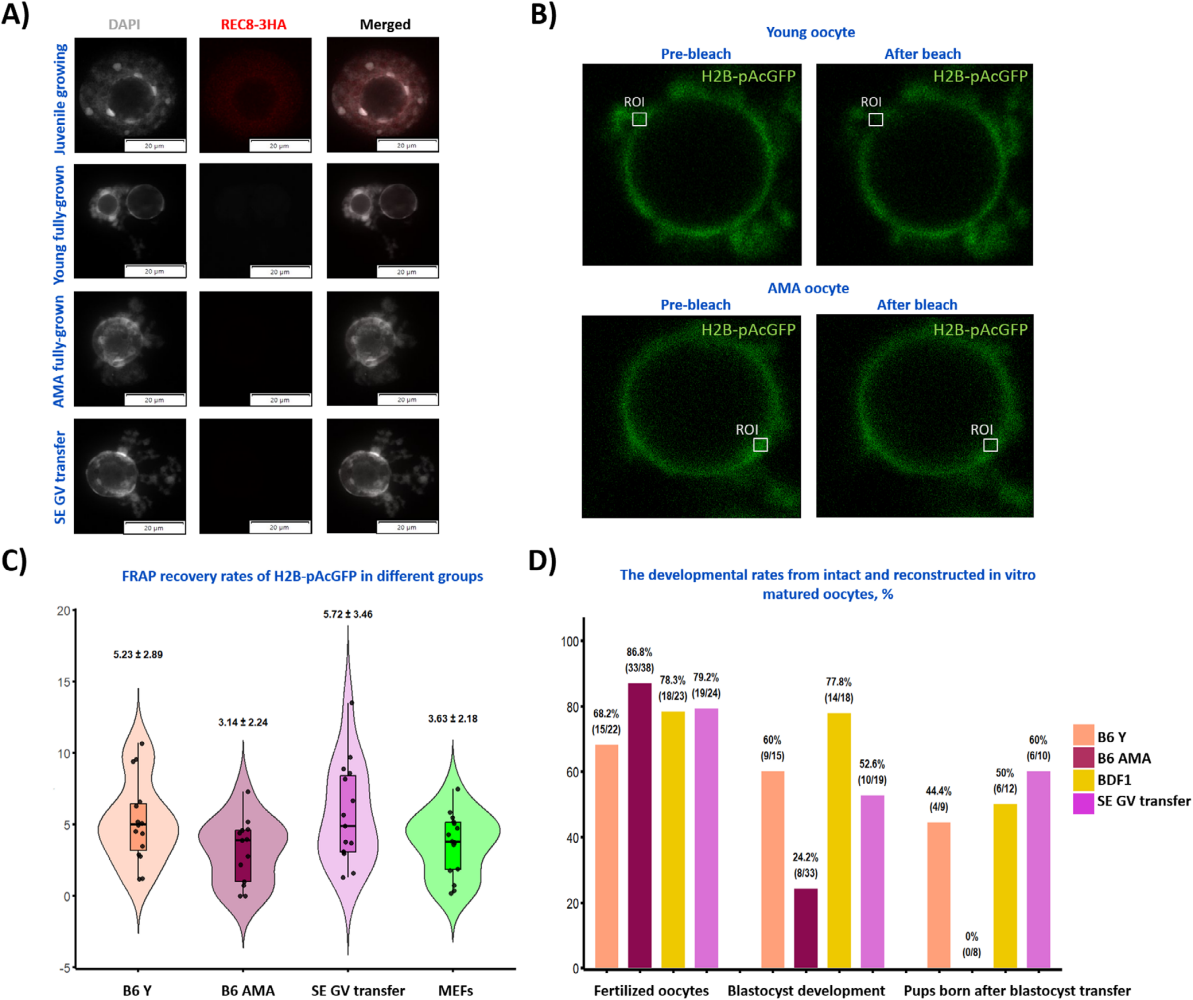
The analysis of chromatin in young and AMA oocytes. (A) Unlike growing oocytes of juvenile animals (positive control), fully grown GV oocytes of both young and AMA animals, and the reconstructed oocytes, do not incorporate exogenous REC8‐3HA. (B) The analysis of chromatin dynamics by FRAP and the typical regions of interest (ROI) chosen. (C) Summary of normalised recovery rates following bleaching. (D) The fertilisation and developmental rates of control and reconstructed oocytes. B6 AMA, oocytes from advanced maternal (AMA) age B6 females; B6 Y, oocytes from young C57Bl/6J (B6) females; BDF1, control recipient strain; MEFs, mouse embryonic fibroblasts; SE‐GV transfer, oocytes reconstructed from cytoplasts obtained by selective enucleation (SE) followed by transfer of the AMA GV.

The loss of REC8 cohesin, typical for mouse AMA oocytes (Chiang et al. 2010), has recently been proposed to lead to persistent DNA damage and reduced repair efficiency upon ectopic DNA damage induction (Sharma et al. 2024). We asked if the same is true for physiologically occurring age‐associated DNA damage. In agreement with previous reports (Tachibana‐Konwalski et al. 2010; Chiang et al. 2010), neither AMA nor young fully grown oocytes noticeably incorporated REC8‐3HA in their nuclei, confirming a limited interaction of REC8‐3HA with chromatin. To check if the incorporation might be stimulated at the damaged sites, we analysed the SE‐AMA GV transfer oocytes. No signal was observed (Figure 2A). Therefore, although the SE cytoplasts effectively suppress the signs of age‐associated DNA damage, this does not seem directly linked to REC8 incorporation or its age‐dependent deterioration.

Next, we asked whether age‐dependent changes to the accessibility of the damaged site could be a factor. To analyse if the chromatin changes with age, we performed fluorescence recovery after photobleaching (FRAP) with H2B‐pAcGFP as a proxy of chromatin looseness (Figure 2B), in oocytes and somatic cells, which are generally regarded as DNA repair competent. In all the samples, recovery was partial. The slowest recovery rates were observed in AMA oocytes (3.14% ± 2.24%), followed by somatic cells (mouse embryonic fibroblasts, MEFs; 3.63% ± 2.19%), oocytes from young animals (5.23% ± 2.89%), and SE‐GV transfer oocytes (5.72% ± 3.46%) (Figure 2C). Despite the apparent trend, a high intercellular variability was observed with the coefficient of variation of > 55% in all groups, including the somatic cells, and the results failed to reach the statistical significance level (young vs. AMA oocytes *p* = 0.056; AMA vs. SE‐GV transfer *p* = 0.065). Still, in addition to suppressing DNA damage, the young karyoplasm partially restores at least some chromatin parameters in the AMA GVs.

Because the SE‐GV transfer oocytes showed an improvement in all the parameters tested, as the final goal, we generated embryos from the reconstructed oocytes and transferred them to recipient females. We used intact young oocytes and intact AMA oocytes as controls. Although we observed lower developmental rates to the blastocyst stage between the young intact oocytes and the SE‐AMA GV reconstructed ones, the rate of pups born was similar between the two groups. By contrast, markedly fewer blastocysts were obtained in the intact AMA group, and no pups were born (Figure 2D).

In summary, our experiments show, for the first time, that age‐dependent changes occur in oocyte chromatin and that the developmental competence of fully grown AMA oocytes can be markedly improved. The GV transfer experiments show that, in general, oocytes can sense DNA damage and repair it effectively, and that this may be closely linked to chromatin accessibility, which is lost with ageing. There are numerous reports supporting this notion in somatic cells, where both ATP‐dependent chromatin remodelling enzymes and the chromatin state have been shown to increase the accessibility of the damage site by unwinding DNA or weakening the interaction between DNA and the histone octamer (for review see Price and D'Andrea 2013; Chen et al. 2024). At the same time, the rise of aneuploidies in AMA oocytes is likely due to the interplay between the nucleus and cytoplasmic factors, as shown by the GV transfers between young and AMA cytoplasm. Although the use of the SE‐GV transfer relies on the availability of donor oocytes and in vitro maturation, which might be the main limiting factors, from a broader perspective, these findings provide a basis for an accessible cell‐based therapy of mammalian oocytes.

## 3 Materials and Methods

Unless stated otherwise, all chemicals were purchased from Sigma Aldrich, Czech Republic. All experiments were repeated at least three times.

### 3.1 Experimental Animals—Mice

All experimental procedures were reviewed and approved by the Animal Welfare Committee of the Institute of Experimental Medicine and by the Czech Academy of Sciences (licence no. 122‐2023‐P). Laboratory mice were obtained from Charles River/Velaz and housed under standard conditions, with a 12‐h light/dark period and water and food *ad libitum*.

### 3.2 Germinal Vesicle (GV) Oocyte Isolation

To obtain oocytes at the GV in order to generate recipient cytoplasts, 8–12 weeks‐old B6D2F1 females were injected with 7 IU of pregnant mare serum gonadotropin (Sergon, Bioveta, Czech Republic) and sacrificed approximately 48 h later. The ovaries were collected in M2 medium supplemented by 2.5 μM Milrinone to prevent the germinal vesicle breakdown (GVBD). The same protocol was applied to 8–12 weeks and 40–44 weeks‐old C57Bl/6J mice. After the ovary isolation, the follicles were punctured, and oocytes were released to M2 media supplemented with Milrinone at a concentration above. Next, the oocytes were collected and transferred into culture media Eagle's MEM, supplemented with 2.5 μM Milrinone, 5% fetal bovine serum, 0.22 mM sodium pyruvate and gentamicin, and cultured for approximately 1 h at 37°C, 5% CO_2_ before the cumulus cells were removed by pipetting.

### 3.3 Metaphase II (MII) Oocyte Isolation

To obtain oocytes at the metaphase II (MII) stage, the mice were injected with 7 IU of pregnant mare serum gonadotropin (Sergon, Bioveta, Czech Republic), which was followed by the injection of 7 IU of human choriogonadotropin (hCG) approximately 48 h later. The oviducts were transferred into M2 media with hyaluronidase, and cumulus‐free oocytes were then transferred to KSOM media before chromosomal spreading.

### 3.4 GV Transfer

The GV transfer was performed using the piezo‐equipped micromanipulator in M2 media, supplemented with 7.5 μg/mL cytochalasin B and 2.5 μM Milrinone at room temperature. After a brief incubation, the GVs were removed by a manipulation pipette from B6D2F1 (recipients) and C57Bl/6J (donors), transferred to a drop containing 1:10 diluted HVJ as instructed by the manufacturer (GenomONE TM‐CF EX, CosmoBio, Japan), and placed under the zona pellucida for fusion. Next, the reconstructed oocytes were transferred to MEM‐based culture media supplemented with milrinone, inspected for fusion, and successfully fused oocytes were washed and transferred to mouse IVM media (MEM supplemented with 5% fetal calf serum, sodium pyruvate and gentamicin).

### 3.5 Selective Enucleation—GV Transfer

The selective enucleation was performed as described (Greda et al. 2006; Gręda et al. 2015). The SE releases the entire soluble GV content as well as NLBs into the oocyte cytoplasm, but the GV membrane with attached chromatin is removed from the oocyte. Then, the GVs were fused to these SE cytoplasts as described above. Reconstructed oocytes were again washed and transferred to the mouse IVM media.

### 3.6 Nucleolus‐Like Body Enriched Cytoplasts—GV Transfer

The nucleolus‐like bodies were removed from B6D2F1 oocytes by enucleolation as described (Fulka et al. 2003) and kept in the manipulation media as above. Next, a different set of B6D2F1 oocytes was used to generate the recipient cytoplasts by removing the whole GV before transferring the B6D2F1 nucleoloplasts and C57Bl/6J GVs under the zona pellucida for HVJ‐induced fusion. The successfully reconstructed oocytes were washed and placed into mouse IVM media.

### 3.7 In Vitro Maturation—IVM

Control intact and reconstructed oocytes were cultured in Eagle's MEM media supplemented with 5% fetal calf serum, 0.22 mM sodium pyruvate, and gentamicin at 37°C in a 5% CO_2_ atmosphere overnight. The next day, the oocytes were inspected for the presence of polar bodies or their remnants. Mature oocytes were either processed for chromosomal spreading or used for in vitro fertilisation.

### 3.8 In Vitro Fertilisation (IVF) and In Vitro Development

For IVF, 8–12 weeks‐old ICR males were used. The IVF procedure was performed essentially as described by Takeo and Nakagata (2011). The cauda epididymis was isolated and placed under the mineral oil; after puncture, the sperm were released into 100 μL of TYH media supplemented with 0.75 mM methyl‐beta‐cyclodextrin and preincubated for 1 h at 37°C, 5% CO_2_. Fertilisation was performed in HTF medium supplemented with freshly prepared reduced glutathione for 5 h at 37°C, 5% CO_2_ atmosphere. Next, the zygotes containing two pronuclei were sorted, washed and transferred to KSOM for further culture.

### 3.9 Non‐Surgical Embryo Transfer—NSET

Pregnant 8–16 weeks old ICR females were used as recipients. This was to ensure that a failed pregnancy does not mask the failed post‐implantation development. The ICR females were mated with ICR males. Plugged females were separated, and transcervical transfer was performed at 2.5dpc as described (Stone 2020). After an initial optimisation of the blastocyst number using control intact IVM‐IVF embryos, the embryos were transferred in batches of 6 per transfer/female.

### 3.10 Chromosome Preparation, Chromosome Aberrations, and Immunofluorescence

The chromosome spreads were prepared as described (Hodges and Hunt 2002). Briefly, the zona pellucida was removed in Acid Tyrode's solution, and samples were transferred to SuperFrost slides (ThermoFisher Scientific, Czech Republic) covered in the spreading solution. The samples were airdried and either stored at −20°C or processed immediately (immunofluorescence). For immunofluorescence, the slides were blocked in phosphate‐buffered saline (PBS) supplemented with 0.1% Triton X‐100 and 1% bovine serum albumin (BSA) for 1 h at room temperature. The primary antibody was diluted in the same buffer, and the slides were incubated at +4°C overnight in a humidified chamber. The next day, the slides were washed in PBS, 3 × 10 min each wash, and incubated with a secondary antibody at 1: 1000 (Donkey anti‐mouse AlexaFluor 488 or 594, ThermoFisher Scientific, Czech Republic) for 1 h at room temperature. The primary antibodies were: anti‐H4K20me3 (Abcam, ab177190, 1: 2000) to aid the chromosome number analysis, anti‐γH2A.X (Abcam, ab26350, clone 9F3 and 1:500), and anti‐γH2A.X (Sigma‐Aldrich, 05–636, clone JBW301, 1: 500) to assess the potential DNA damage, and anti‐CENPA (Cell Signaling Technology, clone C51A7, 1: 500) to detect centromeres. After the final wash, the slides were covered by a drop of ProLong mounting media with DAPI (ThermoFisher Scientific, Czech Republic) and imaged on an Olympus IX73 inverted microscope.

### 3.11 mRNA Preparation and Injection

The template for in vitro transcription was prepared using PCR. The Rec8 was amplified from testes RNA with a forward primer containing the T7 promoter sequence and a 3HA tag reverse primer (T7_mmRec8F: TAATACGACTCACTATAGGaccatgttctactatcctaacgtgcttcagc; mmRec8_3HA_STOP: tcaAGCGTAATCTGGAACATCGTATGGGTAAGCATAATCTGGAACATCATATGGATAAGCGTAATCTGGAACATCGTATGGGTAggggaatttgggtccaggacg). The PCR product was sequenced from the HA tag sequence. The same strategy was used to prepare H2B‐pAcGFP from the pAcGFP N1 plasmid (TaKaRa/Clontech; T7_H2B_F: TAATACGACTCACTATAGGaccatgcctgagccagccaag; pAc_GFP_STOP: ctacttgtacagctcatccatgcc). The in vitro transcription and polyadenylation were performed using the T7 HiScribe kit (New England Biolabs, BioTech) according to the manufacturer's instructions. The GV oocytes were injected in M2 media supplemented with Milrinone as above using the FemtoJet (Eppendorf, Germany).

### 3.12 Fluorescence Recovery After Photobleaching—FRAP

The oocyte samples were injected with H2B‐pAcGFP and incubated for several hours before imaging and FRAP. The timing of the SE‐GV transfer experiment was kept constant, that is, the AMA germinal vesicles were incubated for approximately 4 h in the SE cytoplasts before analysis. The reference somatic cells, 3 T3 clone A31 (European Collection of Authenticated Cell Cultures), were cultured as recommended by the supplier. For the analysis, the cells were transfected using the standard Lipofectamine 3000 (ThermoFisher) protocol with the H2B‐pAcGFP N1 plasmid and taken for FRAP 4 days after transfection. Prior to photobleaching, GV oocytes expressing H2B‐pAcGFP were transferred to a drop of M2 medium with IBMX and 2% PVP (polyvinylpyrrolidone) on a 35 mm microscopy dish and covered with silicon oil. FRAP was performed on the Zeiss 880 confocal microscope with the Airyscan detector. Before bleaching, we acquired two prebleach images of each cell, either somatic (3 T3 A31) or oocyte. Bleaching was performed with a 405 nm laser (35 mW) at 5% laser power on rectangular ROIs with a pixel dwell time of 84 μsec. Recovery was recorded as a time‐lapse series with a 488 nm laser, lasting 900 s, and analysed in FIJI. The StackReg plugin and Linear Stack Alignment with SIFT have been used to mitigate cell movements during image analysis when applicable (Thévenaz et al. 1998; Lowe 2004). The plots for recovery rates were constructed in R Studio.

### 3.13 Statistical Analysis

The percentage of oocytes with chromosomal aberrations and γH2AX positive oocytes in different sample groups was compared by the Fisher exact test. The difference was considered significant for *p*‐value < 0.05, otherwise—not significant (n.s. on the plot). Groups with *p*‐values in the range 0.01–0.05 were marked as “*”, with *p*‐values in the range 0.001–0.01 as “**”, with *p*‐values lower than 0.001 as “***”. The FRAP data analysis was performed using the Mann–Whitney *U*‐test. All statistical analysis was done in RStudio.

## Author Contributions

H.F. designed the experiment, and H.F., T.I. and J.F. performed the micromanipulations. N.D. performed the FRAP, analysed the images and performed the statistical analysis, and N.N. assisted with the FRAP experiment. M.C., P.L., P.B. and R.S. analysed the chromosomal spreads. H.F. and P.B. wrote the manuscript.

## Conflicts of Interest

The authors declare no conflicts of interest.

## Supporting information


**Data S1:** acel70300‐sup‐0001‐supinfo.docx.

## Data Availability

The data that support the findings of this study are available on request from the corresponding author. The data are not publicly available due to privacy or ethical restrictions.
