# Supplementary material for: Oocyte Age‐Dependent DNA Damage Can Be Reverted by the DNA Repair Competent Karyoplasm of Young Oocytes
Source: Aging Cell. 2025 Nov 15;25(1):e70300. doi: 10.1111/acel.70300 (PMC12740095; doi:10.1111/acel.70300)
Supplement: Supplementary file 1 — Data S1: acel70300‐sup‐0001‐supinfo.docx. [file ACEL-25-e70300-s001.docx]

**Supporting information**

**Fig. S1** The average number of oocytes retrieved per female


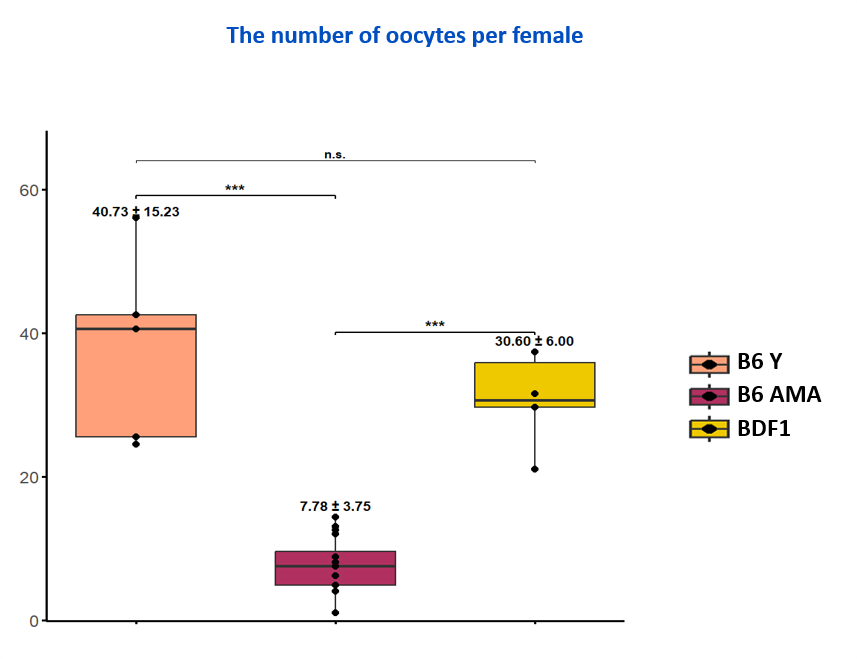


For the young animals, 10 females from at least 5 independent experiments were used to estimate the average number of oocytes. Number of experiments: B6 Y (young) *n=6*; B6 AMA (advanced maternal age) *n=12*; BDF1 *n=5*. For the AMA group, 36 females from 12 independent experimenters were used. The average number of oocytes per female: B6 Y=40.7; B6 AMA=7.8, and BDF1=30.6. The results were evaluated by the Kruskal-Wallis test and Dunn´s test with Bonferroni correction. *** *p*<0.001.

**Fig. S2** The *in vitro* maturation (IVM) rates


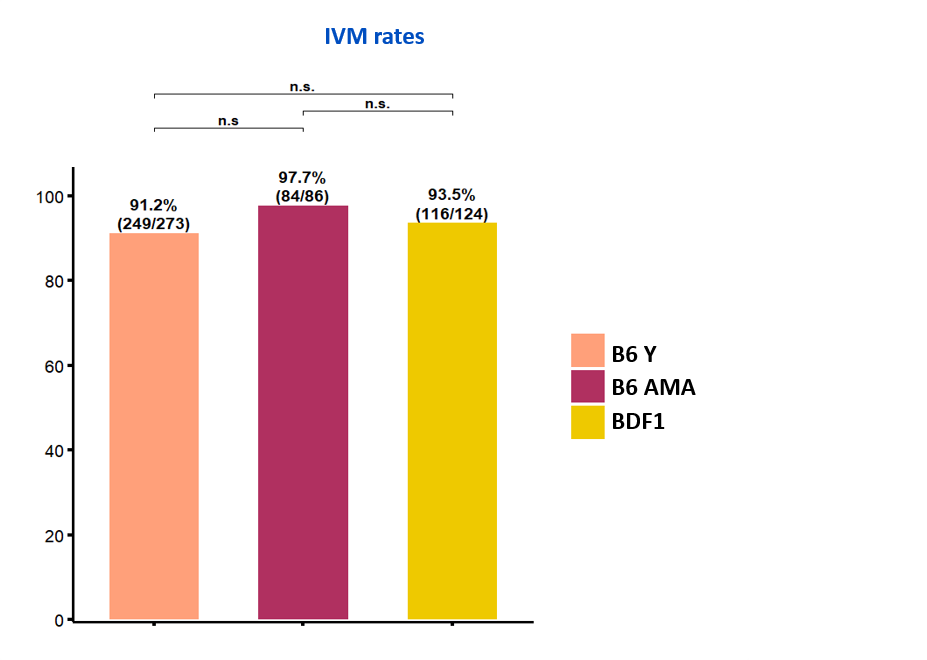


The maturation rates were assessed by the presence of the first polar body or its remnant after an overnight culture and recorded in at least 5 independent experiments for each age group. Number of experiments: B6 Y (young) *n=7*; B6 AMA (advanced maternal age) *n=5*; BDF1 *n=6*. The total number of oocytes analyzed: B6 Y *n=273*; B6 AMA *n=86,* and BDF1 *n=124*. The maturation rates were evaluated by Fisher's exact test (n.s., not significant).

**Fig. S3** The gross nuclear morphology in young and AMA oocytes and the fraction of centromeres associated with nucleoli


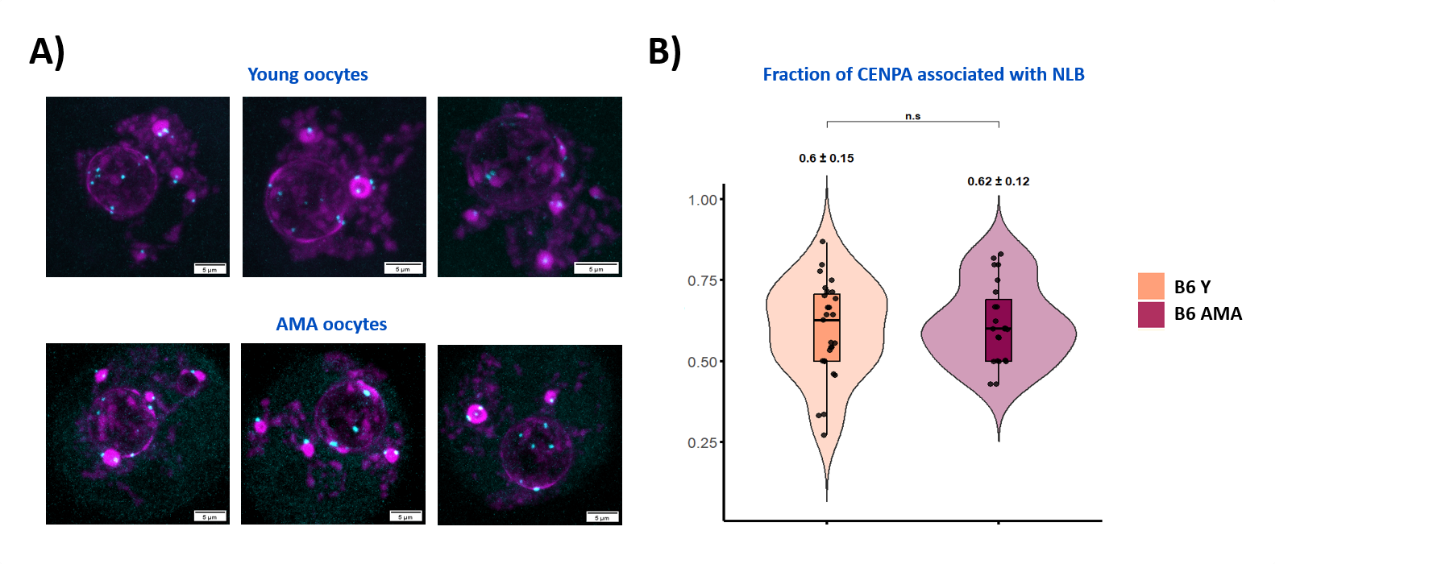


(A) The gross nuclear morphology of oocytes does not appear to be altered with female age and a comparable fraction of centromeres, as detected by CENPA localization (turquoise), is associated with oocyte nucleoli (nucleolus-like bodies). (B) Specifically, 59.6% of all centromeres are associated with nucleolus-like bodies in oocytes from young animals, and 61.6% in AMA oocytes. Total number of oocytes analyzed: B6 Y *n=27*; B6 AMA *n=23*. No chromatin decompaction was observed.

**Fig. S4** The micromanipulations used in the different GV transfer combinations (Related to Fig. 1E)


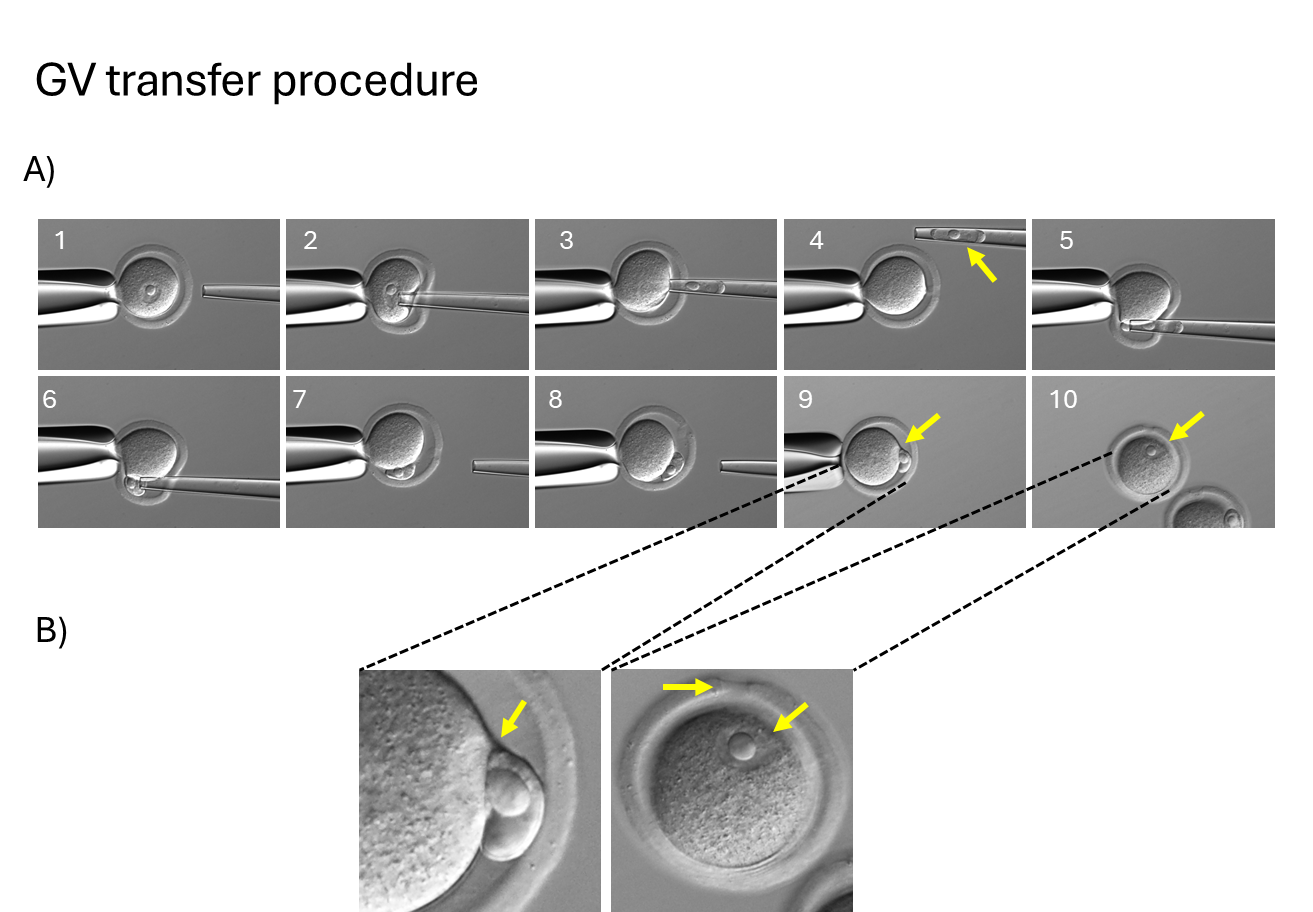


The figure shows a control GV transfer (young GV – young cytoplasm; Y GV – Y cytoplast). (A) First, the zona pellucida is opened using the piezo pipette, which is then used to remove the germinal vesicle. Image 4 shows the complete separation of the cytoplasm (cytoplast) and the germinal vesicle (GV, karyoplast). The karyoplast is then transferred under the zona pellucida of either the same enucleated oocyte (control Y GV- Y cytoplast) or is moved to a different cytoplast (Y GV – AMA cytoplast, AMA GV – Y cytoplast or cytoplast generated by the selective enucleation). (B) Enlarged images 9 and 10 show either the initial fusion between the cytoplast and karyoplast (left, arrow) or the complete fusion. Note the opening in the zona pellucida and the eccentric position of the GV (right, arrows). Essentially, the same procedure was applied to construct the AMA GV – Y cytoplast and the Y GV – AMA cytoplast oocytes.


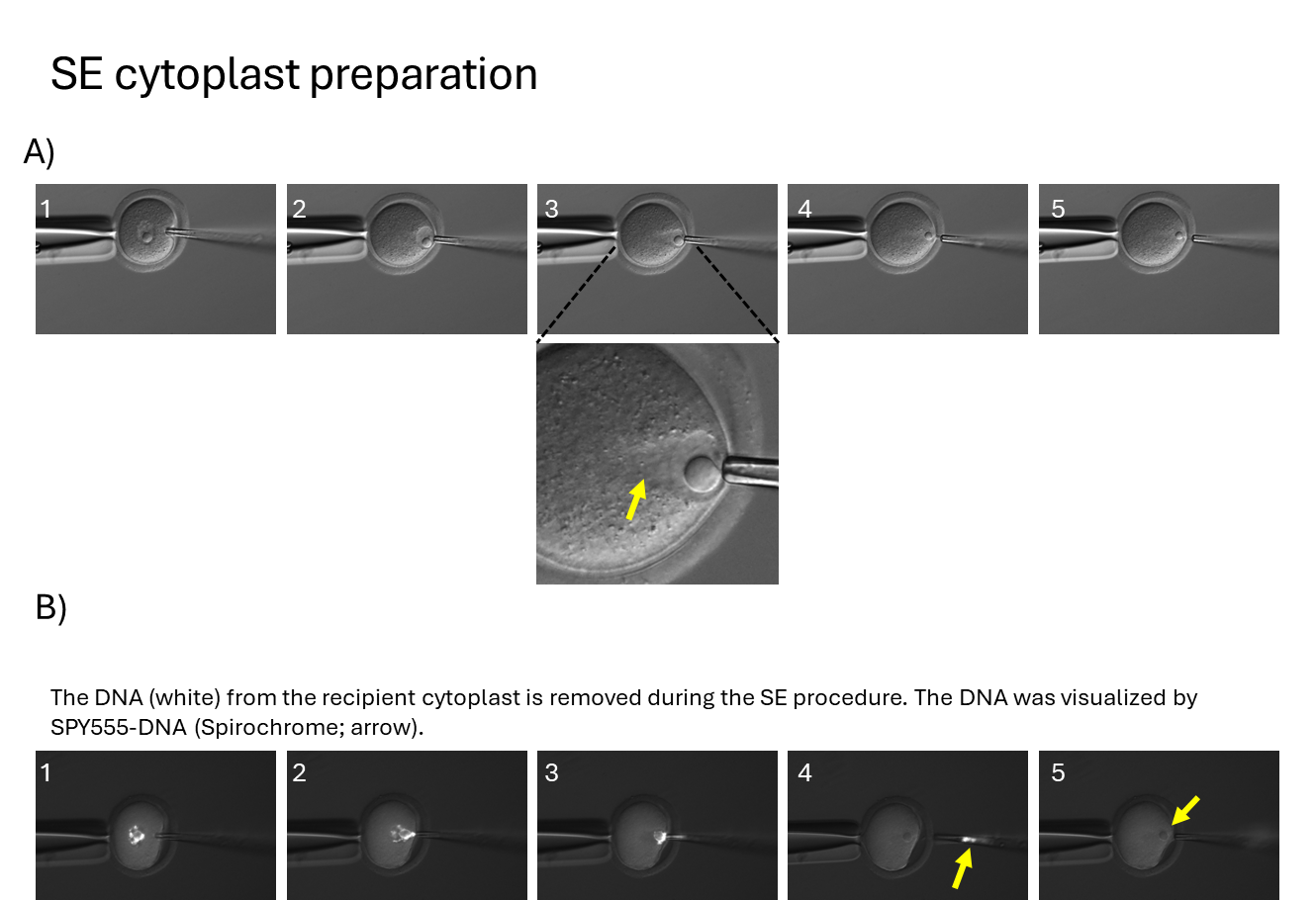


The figure shows the preparation of SE cytoplasts, which were subsequently used as recipient cytoplasts for AMA GVs (SE-GV transfer). For illustration purposes, the DNA in live oocytes was visualized by incubating the oocytes in SPY555-DNA-supplemented manipulation media (50nM). The dye was not used during the main experiments to avoid interference with the immunofluorescence. (A) Image 3 shows the rupture and the spilling out of the soluble nuclear factors (arrow). (B) Image 4 and 5 show the complete removal of the germinal vesicle chromatin, i.e. DNA (Image 4, arrow). Note the nucleolus like body, which remains in the cytoplast (Image 5, arrow).


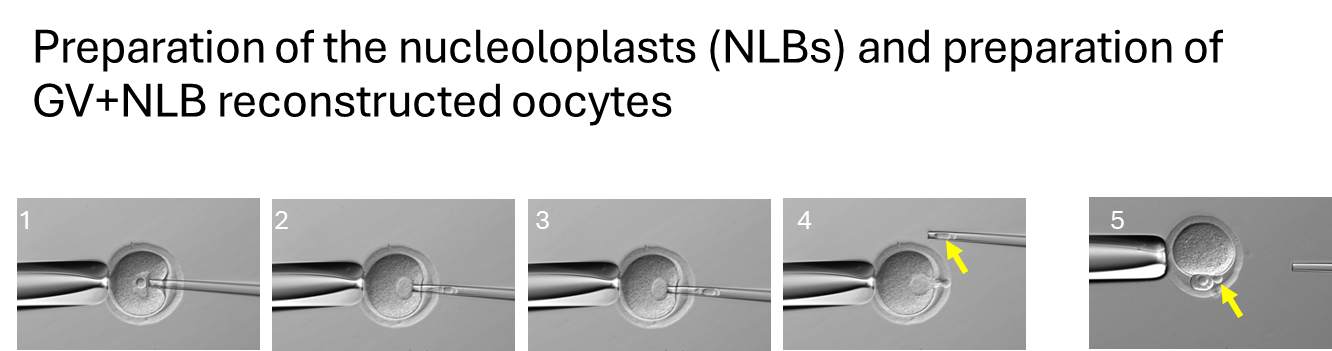


The isolation of nucleoli (nucleolus-like bodies; NLBs) from young oocytes in the form of a nucleoloplast (nucleoli enclosed with the plasma membrane; Image 4, arrow). These nucleoloplasts were combined with cytoplasts generated by a complete enucleation of young oocytes and the AMA GVs, as shown in Image 5 (arrow) (AMA GV – Y NLB – Y cytoplasm). The oocytes with removed NLBs were discarded and not used further.


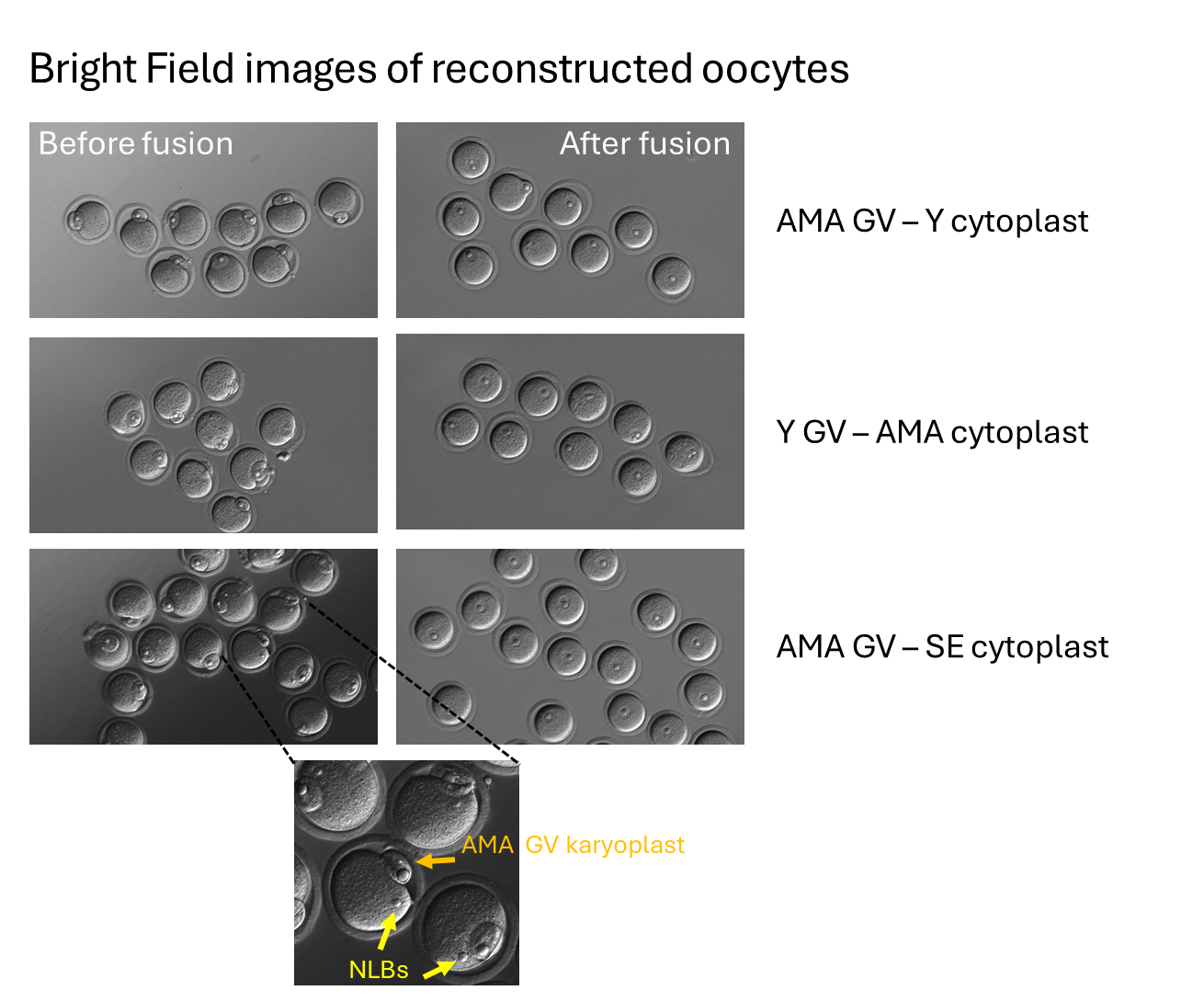


Bright-field images of reconstructed oocytes before and after fusion. AMA GV- Y cytoplast = advanced maternal age (AMA) germinal vesicle (GV) combined with the young (Y) BDF1 cytoplasm; Y GV – AMA cytoplast = young GV combined with AMA cytoplasm; AMA GV – SE cytoplast = AMA GV combined with the cytoplast generated by selective enucleation (SE) of the BDF1 oocyte. Note the presence of release nucleoli (nucleolus-like bodies, NLBs) in the cytoplast (yellow arrow) before fusion with the AMA GV karyoplast (orange arrow).
